# The Lipid Transfer Protein StarD7: Structure, Function, and Regulation

**DOI:** 10.3390/ijms14036170

**Published:** 2013-03-18

**Authors:** Jésica Flores-Martin, Viviana Rena, Sofía Angeletti, Graciela M. Panzetta-Dutari, Susana Genti-Raimondi

**Affiliations:** Universidad Nacional de Córdoba-Consejo Nacional de Investigaciones Científicas y Técnicas, Facultad de Ciencias Químicas, Departamento de Bioquímica Clínica-Centro de Investigaciones en Bioquímica Clínica e Inmunología, X5000HUA Córdoba, Argentina; E-Mails: jflores@fcq.unc.edu.ar (J.F.-M.); vivirena@fcq.unc.edu.ar (V.R.); sofia3677@hotmail.com (S.A.); gpan@fcq.unc.edu.ar (G.M.P.-D.)

**Keywords:** StarD7, START domain, SF-1, β-catenin, ABCG2, cell migration, cell proliferation

## Abstract

The steroidogenic acute regulatory (StAR) protein-related lipid transfer (START) domain proteins constitute a family of evolutionarily conserved and widely expressed proteins that have been implicated in lipid transport, metabolism, and signaling. The 15 well-characterized mammalian START domain-containing proteins are grouped into six subfamilies. The START domain containing 7 mRNA encodes StarD7, a member of the StarD2/phosphatidylcholine transfer protein (PCTP) subfamily, which was first identified as a gene overexpressed in a choriocarcinoma cell line. Recent studies show that the StarD7 protein facilitates the delivery of phosphatidylcholine to the mitochondria. This review summarizes the latest advances in StarD7 research, focusing on the structural and biochemical features, protein-lipid interactions, and mechanisms that regulate StarD7 expression. The implications of the role of StarD7 in cell proliferation, migration, and differentiation are also discussed.

## 1. Introduction

### The START Domain Protein Family

Lipids are currently recognized as versatile and dynamic regulators of various cellular processes such as growth, development, survival, intracellular signaling, and membrane trafficking. Lipid alterations are present in most human diseases such as cardiovascular disease, obesity, metabolic defects at birth, cancer, Alzheimer’s disease, and preeclampsia. The transport of lipids and proteins between organelles is a crucial event in the organization of different cellular compartments. This process is mediated by vesicular transport through the fusion of vesicles to an acceptor compartment or by monomeric transport among different organelles [1]. Monomeric exchange requires specific proteins that stimulate lipid exchange between cell membranes. Several structurally different intracellular protein families that can transport lipid monomers across the aqueous phase have been reported [2]. Among these, the steroidogenic acute regulatory protein-related lipid transfer (START) domain superfamily constitutes a family of proteins that is evolutionarily conserved and widely distributed in nature [3–5]. The proteins share sequence similarity in a 210-amino-acid globular domain that has been implicated in lipid and sterol binding [6,7]; this domain is present in bacteria, plants, protists, yeast, and animals but not in archaea [4,5,8–10]. The typical START domain folds into a helix-grip structure with α-helices at the amino and carboxy termini separated by 9 β-sheets and 2 α-helices forming a hydrophobic pocket for binding sterols and other lipids [6,11]. Based on their phylogenetic relationships, the 15 well-characterized mammalian START domain-containing proteins are grouped into six subfamilies [3,12]. The first member of the family to be reported was the steroidogenic acute regulatory protein (StAR/StarD1), which transfers cholesterol to the mitochondria in steroid-producing cells [13]. This protein gives name to subfamily 1, which includes another cholesterol-binding protein, StarD3/MLN64 [14]. The other subfamilies include the cholesterol- and oxysterol-binding proteins StarD4, StarD5, StarD6; the StarD2/PCTP, StarD7, StarD10, and StarD11/CERT subfamily that binds phospholipids and sphingolipids; the StarD8/DLC-3, StarD12/DLC-1, and StarD13/DLC-2 subfamily (with Rho-GTPase signaling function); the StarD14/ACOT11 and StarD15/ACOT12 subfamily (with thioesterase activity), and the StarD9/Kif16 subfamily. To date, the crystal structures of hStarD1, hStarD3, mStarD4, hStarD5, hStarD2/PCTP, StarD11/CERT, hStarD13, and hStarD14 START domains have been reported [5,7,15–21].

In this review, we summarize the current state of knowledge in StarD7 research, focusing on the molecular characteristics, protein-lipid interactions, and mechanisms that regulate StarD7 expression.

## 2. Structural and Biochemical Characteristics of StarD7

**Figure 1:**
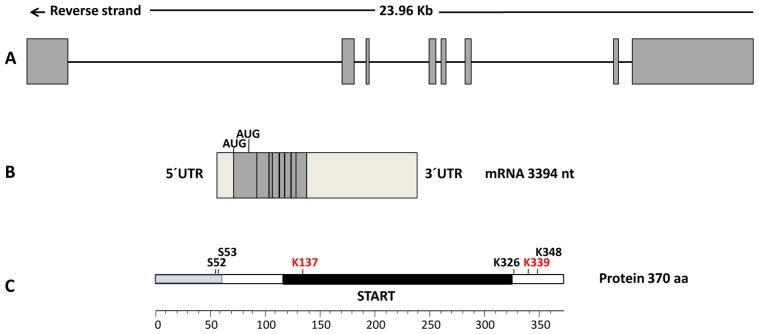
Schematic representation of the human StarD7 gene, mRNA, and protein. (**a**) Gene organization. The relative position and approximate sizes of eight exons spanning 23.96 kb on chromosome 2 is shown in a linear representation; (**b**) mRNA (NM_020151.3) schema. Putative translation initiation AUG codons encoding the StarD7-I of 370 (positions 402–1514) and StarD7-II of 295 (positions 627–1514) amino acid residues are shown. Light grey boxes represent 5′UTR and 3′UTR regions; (**c**) The StarD7 protein (370 aa). Putative ubiquitination (red) and phosphorylation (black) sites as well as the mitochondrial localization signal (grey box) and the steroidogenic acute regulatory protein-related lipid transfer (START) domain (black box) are indicated.

The StarD7, located on the short arm of chromosome 2p12-2p11.2, comprises 8 exons spanning 23.96 kb (Figure 1). StarD7 mRNA (first designated GTT1, accession number AF270647.1) was originally identified by differential display techniques as a transcript that had higher expression in the choriocarcinoma cell line JEG-3 than in normal and benign trophoblastic samples. This original mRNA encodes a protein of 295 amino acid residues (accession number AAF81750.1) with a molecular weight of approximately 34.7 kDa and a pI of 5.79 [22], with 25% identity and 49% similarity in amino acid sequence with the human, bovine, and mouse phosphatidylcholine transfer protein (PCTP) also known as StarD2 [23]. The updated StarD7 mRNA reference sequence (NM_020151.3) encodes a protein of 370 amino acid residues (positions 402–1514) with a theoretical molecular weight of 43.1 kDa and a pI of 9.04. The mRNA sequence has a second in-frame putative translation initiation AUG codon position 627 from which the short version of protein is possibly encoded. This short StarD7 type is predicted to localize in the cytoplasm, whereas the larger StarD7 type contains an additional 75 amino acid stretch with a putative mitochondrial localization signal at the *N*-terminal region. Our group provided the first evidence of the presence of the StarD7 protein in human trophoblast cells, revealing a unique specific band of approximately 34 kDa, which suggested that the predicted StarD7 34.7 kDa peptide and not the 43.1 kDa peptide is synthesized in human trophoblastic tissues [24]. StarD7 was detected by mass spectrometry and immunoblot analysis as a 35 kDa protein in human normal testes [25]. Recently, Horibata and Sugimoto detected 2 protein bands in lysates prepared from cells transfected with an expression vector for the larger StarD7 type, StarD7-I [26]. They proposed that StarD7 is synthesized as a 43 kDa precursor that is processed into an approximately 33 kDa mature form by cleavage of the mitochondrial-targeting sequence. The overexpressed StarD7-I localized mainly in the mitochondria of mouse hepatoma HEPA-1 cells cultivated at low cellular density, but was present in the cytoplasm of cells cultivated at high cellular density. In contrast, the overexpressed short-type form (StarD7-II) was distributed in the cytoplasm at all cellular densities. Notably, the molecular weight of endogenous StarD7 detected in both the cytoplasm and mitochondria was approximately 33 kDa, which was the same as the molecular weight of the mature form of StarD7-I and also StarD7-II [26].

Two ubiquitination sites at lysine 137 and lysine 339 of StarD7 (NP_064536.2) were identified by affinity capture and mass spectrometry [27]. In addition, 4 potential phosphorylation sites at serine 52, serine 53, lysine 326, and lysine 348 have been predicted [28].

Far-ultraviolet circular dichroism spectroscopy shows that the StarD7 spectrum has a minimum at 218 nm, which is typical of a predominantly β-sheet conformation [29]. Secondary structural characteristics indicate that the helical count of StarD7 is not similar to that of known crystal structures of StarD3 and StarD4. Also, StarD7 did not elicit a steroidogenic response when it was added to isolated pig adrenal mitochondria and pregnenolone synthesis was measured [29]. A study using an oriented peptide array library approach predicted that the sequence located between amino acids 152 and 167 of the StarD7-II protein might be a protein kinase A (PKA) target, suggesting that StarD7 is a phosphoprotein that is regulated by the cyclic adenosine monophosphate (cAMP)/PKA pathway [30]. StarD7 was one of 139 protein candidates involved in interactions with the tuberous sclerosis complex identified by tandem mass spectrometry analysis [31].

In summary, current data support that StarD7 is synthesized as a short-lived precursor protein of 43.1 kDa and is processed to a 34.7 kDa mature protein that can undergo posttranslational modifications that modulate its functionality.

## 3. StarD7-Lipid Interaction

Several studies have shown that StarD7 interacts, binds, and transports lipid molecules. An earlier study demonstrated that StarD7 is a surface-active protein, since it forms stable monolayers by adsorption at the air-buffer interface [32]. StarD7 injected into the subphase solution readily penetrated and spread at lipid monolayers, as indicated by an increase in the surface pressure of the system. The maximum surface pressure reached varied by the lipid present in the monolayer: High interfacial stabilization for phosphatidylglycerol, dilaurylphosphatidylcholine, phosphatidylserine (PS), and cholesterol; intermediate interfacial stabilization for dipalmitoylphosphatidylcholine; and relatively low interfacial stabilization for sphingomyelin (SM). These findings indicate that the surface activity of StarD7 is strong enough to thermodynamically drive and retain the protein at the lipid-membrane interface, where it may undergo lipid-dependent reorganization, as indicated by changes in surface pressure and electrostatics [32].

The mechanism of StarD7 interaction with the lipid-membrane interface has been elucidated by fluorescence dequenching of small unilamellar vesicles (donor liposomes) labeled with 2-(4,4-difluoro-5, 7-dimethyl-4-bora-3a,4a-diaza-s-indacene-3-pentanoyl)-1-hexadecanoyl-sn-glycero-3-phosphocholine (BODIPY-FL-C5-HPC) by dilution with nonlabeled large unilamellar vesicles (acceptor liposomes) [33]. This approach demonstrated that recombinant StarD7 accelerates the dilution of BODIPY-FL-C5-HPC in a concentration-dependent manner. Therefore, StarD7 may facilitate close apposition of membranes and initiate membrane aggregation. Further, fluorescence energy transfer analysis, liposome size distribution analysis, and the multinuclear giant cell formation induced by recombinant StarD7 strongly indicate that StarD7-induced lipid dilution occurs via bilayer fusion. This process is favored by phosphatidylethanolamine (PE), which is known to stabilize the nonlamellar phases, which are considered intermediary structures in the fusion process. Collectively, these data support that StarD7 plays an important role in lipid intermembrane traffic by promoting membrane fusion [33].

In addition to the StarD7-lipid interaction, our group demonstrated by *in vitro* ELISA binding assays that the StarD7 protein can bind cardiolipin and PS but not ceramide or phosphatidylinositol (unpublished results). In addition, fluorescence resonance energy transfer-based assays have shown that StarD7-I has a much higher preference for phosphatidylcholine (PC) than for PS, PE, or SM (approximately 5% of its PC-transfer activity) [26]. A comparison of phospholipid ligand specificities of StarD7-I and StarD7-II revealed that both proteins have a preference for PC, although the specific activity of StarD7-I is slightly greater than that of StarD7-II. Horibata and Sugimoto concluded that StarD7 facilitates the delivery of PC to the mitochondria, and suggested that StarD7 extracts PC from the cytoplasmic surfaces of the endoplasmic reticulum (ER), Golgi apparatus, or plasma membranes [26]. In line with the membrane association and transporter ability of StarD7, our group demonstrated that StarD7 shows a partial relocalization to the plasma membrane in *in vitro* differentiating cytotrophoblast cells, supporting that StarD7 plays a role in the delivery of lipids to the plasma membrane [24]. More recently, it has been reported that PC can reach or leave lipid droplets by various mechanisms, one of which could involve the transporter capacity of StarD2, StarD7, and StarD10 proteins [34]. In addition to the monomeric nonvesicular mechanism, lipid transfer proteins have been implicated in a more efficient transport mechanism that involves phospholipid exchange between the ER and other organelles (e.g., mitochondria, lipid droplets, endosomes, Golgi apparatus, plasma membrane) at specialized regions of the ER called membrane contact sites [1,2,26,35]. On the basis of these observations and the findings of Angeletti *et al.*, it can be proposed that StarD7 contributes to lipid intermembrane exchange by promoting transient hemifusion [33]. Together, these observations support that StarD7, as a member of the START domain proteins, facilitates lipid nonvesicular transport between membranes.

## 4. StarD7 Expression

Human StarD7 orthologous genes have been annotated in the Ensembl and GenBank databases in different genomes, suggesting a conserved physiological function. In addition, the StarD7 protein has been predicted in most animal phyla, vertebrates, and invertebrates as well as in plants, which underscores its functional role.

Semiquantitative RT-PCR assays in a series of tumor cell lines have shown that StarD7 has widespread expression, predominantly in trophoblast-derived JEG-3, JAR, and HTR8-SVneo cells, hepatocellular carcinoma HepG2 cells, and colorectal adenocarcinoma HT29 and Caco-2 cells. Low StarD7 transcript levels are found in human cervix adenocarcinoma HeLa cells, human breast adenocarcinoma MCF7 cells, human lung adenocarcinoma A549 cells, human melanoma SK-MEL-31 cells, human acute myelocytic leukemia K-562 cells, and human promyelocytic leukemia HL-60 cells [22]. Western blot and immunohistochemical assays show that the StarD7 protein is present in the cytotrophoblast and syncytiotrophoblast layers of normal-term and early placentas, complete hydatidiform mole, and choriocarcinoma tissue, as well as in the above-mentioned cell lines [24]. Interestingly, StarD7 is a target gene of Has-miR-377, which is downregulated in the preeclamptic placenta, suggesting that StarD7 expression may be altered in preeclampsia [36]. Several genome wide analyses have shown that StarD7 is differentially expressed in various cell and tissue samples under different experimental conditions such as metabolic state, inflammation, cancer, and behavioral changes (Table 1). These studies suggest that StarD7 transcript levels are tightly controlled in normal cell physiology.

## 5. Transcriptional Regulation of StarD7

### 5.1. Regulation of StarD7 Expression by β-Catenin/T Cell-Specific Transcription Factor 4

**Figure 2:**
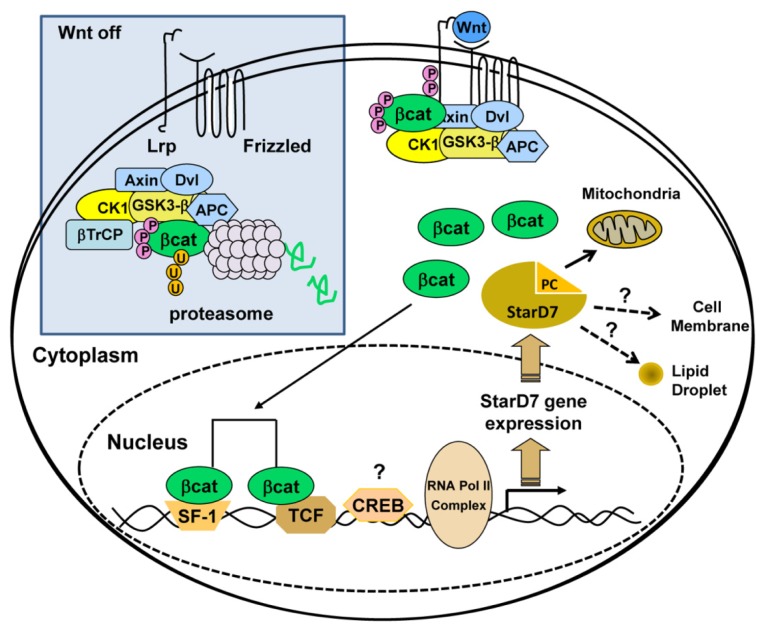
Steroidogenic factor 1 (SF-1)/β-catenin upregulates StarD7 expression in JEG-3 cells. The proposed model shows SF-1/β-catenin mechanisms involved in StarD7-induced expression, based on current data. The Wnt/β-catenin pathway is a conserved cell-cell signaling mechanism in animals that regulates gene expression via the TCF/LEF1 family to coordinate many cellular processes. In the absence of Wnt signaling, the destruction complex remains in the cytoplasm, where it binds, phosphorylates, and ubiquitinates β-catenin via the β-transducing repeat-containing protein (βTrCP). Finally, the proteasome recycles the complex by degrading β-catenin (Wnt off). In the presence of Wnt signaling, the destruction complex captures and phosphorylates β-catenin but ubiquitination by β-TrCP is blocked. This results in accumulation and nuclear localization of the newly synthesized β-catenin. In the nucleus, β-catenin regulates target gene expression by interacting with TCF/LEF1 transcription factors [69]. Free β-catenin interacts with SF-1 to increase StarD7 transcription [65]. In addition, the cAMP response element-binding protein (CREB) may bind putative cAMP response elements that modulate gene expression. StarD7 mediates PC intracellular trafficking to the mitochondria [26] and possibly to the plasma membrane [24] and lipid droplets [34]. APC, adenomatous polyposis coli; CK1, casein kinase 1; Dvl, Dishevelled; LrP, low density lipoprotein receptor-related protein.

The first evidence of the regulation of StarD7 by β-catenin/T Cell-Specific Transcription Factor 4 (TCF4) was given by Lee *et al.*[54], who reported that StarD7 was among the 33 Wnt-dependent candidate genes in hepatoma cell lines. The Wnt/β-catenin pathway is a conserved cell-cell signaling mechanism in animals that regulates gene expression via the TCF/lymphoid enhancer-binding factor 1 (LEF1) family to coordinate many cellular processes such as proliferation, differentiation, and cell motility in normal development and cancer cell progression [55]. Genes encoding Wnt-signaling factors are expressed in the human placenta and in different trophoblast cell models such as JEG-3 cells, consistent with their ability to send, receive, and inhibit Wnt signals [56–58]. In line with this, numerous reports have highlighted the role of the Wnt signaling pathway in implantation, placentation, and trophoblast differentiation [56–63]. β-Catenin and TCF4 regulate expression of StarD7 by interacting with its promoter region. Moreover, glycogen synthase kinase 3β (GSK3β) inactivation leads to β-catenin stabilization and translocation into the nucleus, resulting in increased StarD7 mRNA and protein levels as well as increased promoter activity [64]. Site-directed mutagenesis of the TCF4 motif, located −614/−608 bp relative to the StarD7 transcription start site, markedly diminishes its promoter activity. Importantly, the interaction of TCF4 and β-catenin with the human StarD7 promoter has been confirmed *in vivo* by chromatin immunoprecipitation (ChIP) assays. Taken together, these studies strongly indicate that Wnt signaling regulates StarD7 transcription in JEG-3 cells through the canonical pathway [64] (Figure 2).

### 5.2. Regulation of StarD7 Expression by cAMP and Steroidogenic Factor 1

In addition to the TCF4-binding site, which is required to activate the StarD7 promoter by the β-catenin/TCF4 transcription factor [65], 3 potential binding motifs have been identified for the orphan nuclear receptor steroidogenic factor 1 (SF-1): A sequence at −792/−785 (CAAGGTCA, upper strand) and 2 other potential binding sites, the sequences at −493/−486 (CAAGGACA, upper strand) and at −169/−162 (CTACCTTG, lower strand). Four putative cAMP response elements (−510/−489, −235/−214, −160/−139, and −116/−96) have also been identified.

SF-1 is a member of the nuclear receptor family that plays multiple roles in development and metabolism. This transcription factor, identified in all steroidogenic tissues, including the placenta, is required for the differentiation of mammalian endocrine glands and for sexual development [66,67]. SF-1 plays a role in controlling the expression of several cAMP-responsive genes such as the human StAR/StarD1 [68]. In support of this, our group reported that SF-1 overexpression and cAMP addition have additive effects on StarD7 promoter activity. Deletion analysis of the StarD7 promoter region has revealed that the −792/−785 SF-1-1 consensus binding is required for SF-1-mediated transcriptional induction. This effect is also increased by the addition of forskolin, suggesting the involvement of PKA activation [65].

Electrophoretic mobility shift assays and competition analysis have shown that SF-1 can associate with the StarD7 promoter region mainly through the SF-1-1 (−792/−785) motif. However, disruption of this binding site led to an approximately 30% decrease in StarD7 reporter activity, suggesting that SF-1 regulates StarD7 expression through diverse promoter interactions in addition to this *cis*-element [65].

### 5.3. Cross-Talk between β-Catenin/TCF4 and SF-1 Pathways

There is increasing evidence to show that the nuclear hormone receptor and canonical Wnt pathway interact at different levels to regulate cell growth, proliferation, differentiation, apoptosis, and metastatic potential in various tissues [70]. β-Catenin acts as a coactivator of SF-1 when it transduces Wnt signals to Dax1 [71], StarD1 [72], aromatase [73]; GnRH receptor [74], Müllerian inhibiting substance type II receptor [75], LHB/Lhb [76], and inhibin [77] target genes. Consistent with this, ChIP and site-directed mutagenesis assays have shown that activation of StarD7 expression requires the binding of β-catenin to the TCF4 transcription factor, suggesting that β-catenin functions as a bridge between SF-1 and TCF4 to form a ternary complex, which in turn activates StarD7 expression [66].

Collectively, these findings indicate that β-catenin acts in conjunction with SF-1 to activate the StarD7 promoter (Figure 2). The autocrine and paracrine actions of Wnt signaling in combination with SF-1 on StarD7 expression may have important implications on phospholipid uptake and transport, contributing to the normal development of trophoblast cells.

## 6. StarD7 Modulates Trophoblast Physiology

### 6.1. *StarD7* Modulates ATP-Binding Cassette Subfamily G (WHITE) Member 2 Expression

In addition to intracellular lipid transport, START proteins may play various other key biological roles. StarD2/PCTP is involved in energy substrate utilization [78]; StarD12/DLC-1, StarD13/DLC-2, and StarD8/DLC-3 are present at low levels or are absent in several human cancer tissues, suggesting their contribution in tumorigenesis [79,80]; and StarD10 is overexpressed in breast cancer and cooperates with ErbB receptors in cellular transformation [81]. To determine the other potential roles of StarD7, siRNA assays have been used to silence StarD7 expression in JEG-3 cells. Exploratory differential gene expression analysis shows that the ATP-binding cassette subfamily G member 2 (ABCG2) is one of the most abundantly downregulated mRNAs [82]. Immunofluorescence assays showed that the ABCG2 protein localized to the plasma membrane of JEG-3 cells treated with scrambled siRNA, but the immunofluorescent ABCG2 signal is low or absent in StarD7 siRNA-treated cells [83].

ABCG2 is a member of the ABC protein superfamily of multidrug efflux transporters [84]. ABCG2 is an integral plasma membrane glycoprotein distributed in normal human tissues and highly expressed in those with barrier function, such as the placenta, testes, liver, kidney, intestine, and brain [85]. Besides its role as a drug and xenobiotic transporter or in protection of the fetus against potential toxicity [86,87], other physiological functions of ABCG2 in the placenta have been proposed [85]. ABCG2 may play a role in the transverse distribution of lipids in the plasma membrane during trophoblast syncytialization [88]. *In vitro* trophoblast fusion and differentiation are accompanied by a significant increase in ABCG2 expression [89], whereas inhibition of ABCG2 activity causes cytokine-induced trophoblast cell apoptosis [90]. Low expression of ABCG2 in the placenta occurs in intrauterine growth retardation, suggesting that a decrease in ABCG2 may cause a deficit in placental function and survival [88,90]. Therefore, low StarD7 expression might also be related to placental dysfunction.

### 6.2. StarD7 Modulates Cell Migration, Proliferation, and Differentiation

StarD7 knockdown causes an increase in β-human chorionic gonadotropin (βhCG) mRNA expression as well as protein synthesis and secretion [83]. There was concomitant induction of the endogenous syncytin-1 mRNA level and a slight but significant reduction of intercellular desmosomes between adjacent JEG-3 cells [83]. Thus, downregulation of StarD7 induced trophoblast cell fusion and a higher expression of biochemical differentiation markers. There was also a significant reduction in cell migration, cell proliferation, and phospholipid biosynthesis in StarD7 siRNA silenced JEG-3 cells [83]. Preliminary studies have shown similar results in other epithelial cell lines [91], suggesting a conserved function of StarD7 in cell physiology.

**Figure 3:**
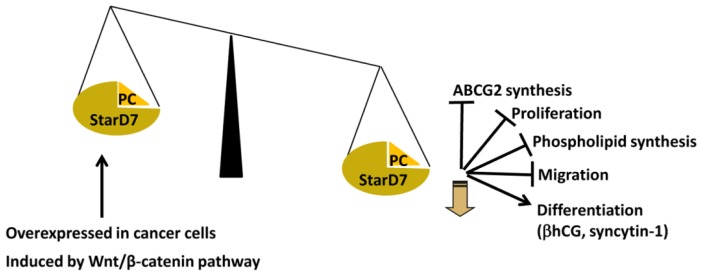
The role of StarD7 in various cellular events in JEG-3 cells. StarD7 knockdown causes a reduction in ABCG2 and phospholipid synthesis, decreased cell migration and proliferation, and increased βhCG and syncytin-1 transcription [83]. High StarD7 levels have been found in several types of cancers (Table 1). StarD7 is induced by the Wnt/β-catenin pathway [64].

Although downregulation of StarD7 induces differentiation of JEG-3 cells, StarD7 expression increases in normal cytotrophoblast cells undergoing spontaneous *in vitro* syncytialization [24]. Thus, it appears that an optimal StarD7 level is required to maintain normal cell physiology (Figure 3). Several reports support this assumption. First, StarD7 was originally found to be upregulated in the choriocarcinoma JEG-3 cell line as compared with the nonmalignant counterpart, complete hydatidiform mole, and normal trophoblastic tissue [22]. Second, StarD7 is overexpressed in several types of cancer (Table 1). Third, the StarD7 promoter is activated by the Wnt/β-catenin signaling pathway [64], which promotes proliferation and is frequently altered in cancer cells [38]. In contrast, the induction of βhCG synthesis and secretion after StarD7 silencing is consistent with the finding that high hCG production and secretion are associated with several pathological alterations in syncytiotrophoblast function [92,93]. These results indicate that dysregulation of StarD7 expression could result in altered trophoblast function or differentiation, leading to an increased risk of placental disorders. These findings provide evidence for a new role for StarD7 in trophoblast cell physiology. Further investigations are needed to elucidate the mechanisms involved in these events.

## 7. Conclusions

StarD7 is a member of the START domain superfamily, which is involved in various physiological processes such as lipid transfer, metabolism, and modulation of signaling pathways. Since its discovery, the biochemical characteristics and lipid interaction properties of StarD7 have been described. The expression of StarD7 has been shown in several human cell lines and in some human and mouse tissues. Genome wide analysis has shown that the StarD7 transcript is altered in various conditions such as metabolic state, inflammation processes, cancer and behavior. The regulation of StarD7 expression in trophoblast cell lines by Wnt/β-catenin and SF-1 has also been reported.

An important question that remains unanswered is whether the biological functions of StarD7 are related only to phosphatidylcholine delivery to mitochondria or can be extended to other intermembrane lipid transfer activities.

Despite the well-established role of StarD7 in modulating trophoblast cell proliferation, migration, and differentiation, the mechanisms that control these processes remain largely unknown. Elucidating these mechanisms is important to understand several pathologies that are associated with pregnancy, such as recurrent miscarriage, gestational diabetes, intrauterine growth retardation and preeclampsia, in which trophoblast cell proliferation, migration, and differentiation are altered.

## Figures and Tables

**Table 1:** Differential expression of StarD7 in cells and tissues as determined by genome wide analysis. *

Physiological/Pathological Major Situations	Study	Cells/Tissue	Fold Change	Expression	Reference
Cancer	Ductal carcinoma *in situ vs.* normal breast tissue	Myoepithelial cells	3	Upregulated	[37]
	B-chronic lymphocytic leukemia *vs.* normal B cells	Peripheral blood mononuclear cells	>97.7	Upregulated	[38]
	Microdissected tumor cells	Colorectal tissue	2.59	Upregulated	[39]
	MIA PaCa-2 cells transfected with miR-193b	Pancreatic cell lines	2	Downregulated	[40]
	HCT-C18 cells treated with 5-fluorouracil	Human colon cancer cell lines	2	Upregulated	[41]
Inflammation	Chronic periodontitis *vs.* normal	Peripheral blood neutrophils	2.19	Upregulated	[42]
	Conventional M1 macrophages treated with oxidized phospholipid	Macrophages	3.02	Upregulated	[43]
	Hypersensitivity pneumonitis *vs.* idiopathic pulmonary fibrosis	Lung	ND **	Upregulated	[44]
	Human idiopathic dilated cardiomyopathy *vs.* nonfailing	Heart	0.62	Downregulated	[45]
Metabolic state	Quantitative trait loci on chromosome 2 associated with growth and fatness	Liver	1.17	Upregulated	[46]
	Short-term high-fat-diet-fed mice	Skeletal muscle	1.3	Upregulated	[47]
	Insulin resistance *vs.* insulin sensitive	Omental adipose tissue	0.28	Downregulated	[48]
	Fasted mice	Small intestine	1.4	Upregulated	[49]
	Hyperinsulinemic clamp	Skeletal muscle	3.31	Upregulated	[50]
	Lengthening *vs.* shortening contraction	Leg muscle biopsies	2	Downregulated	[51]
Behavior	Conditional fear	Brain	ND **	Upregulated	[52]
	Singing *vs.* nonsinging songbird behavior	Forebrain vocal nuclei of brain	DIH **	Upregulated	[53]

* StarD7 transcript expression levels are grouped according to the physiological or pathological major situations studied.

** ND: Not determined; DIH: Detected by *in situ* hybridization.
